# Digital breast tomosynthesis improves the accuracy of the diagnosis of circumscribed lesions because of increase of margin visibility

**DOI:** 10.1186/bcr3701

**Published:** 2014-11-03

**Authors:** R Wasan, J Morel, A Iqbal, D Evans, J Goligher, C Peacock, R Rahim, K Satchithananda, M Michell

**Affiliations:** 1 King's College Hospital, London, UK

## Introduction

Circumscribed masses are a common cause of recall for assessment but most are benign. The aim of this study is to compare the accuracy of **digital breast tomosynthesis (DBT) **with digital mammography (DM) in assessing the margin of circumscribed lesions as a predictor of benign or malignant disease.

## Methods

The study group consisted of women recalled from breast screening for further assessment of circumscribed masses. Clients underwent co-registered DM and DBT in both MLO and CC projections. Two experienced breast radiologists evaluated DM and DBT images and a consensus decision was reached on the percentage of the margin that was well defined on DM and DBT. The lesions were categorised 1 to 4 as follows: 1 = 0 to 25%, 2 = 26 to 50%, 3 = 51 to 75% and 4 = 76 to 100%.

## Results

One hundred and twenty circumscribed lesions were evaluated. Data on 118 lesions seen on the MLO view are presented. There were 93 benign lesions and 25 cancers. There was a change in distribution of margin categories between DM and DBT. More lesions were categorised as 3 or 4 on DBT (59/118) compared with DM (18/118). Of the 93 benign lesions, 17 were categorised as 3 or 4 on DM and 57 on DBT. The difference between the two proportions was significant (*P *< 0.0002). There were more cancers categorised as 1 on DBT (21/39 = 54%) compared with DM (20/76 = 26%); Fisher's exact test (*P *< 0.004).

## Conclusion

Increased margin visibility of circumscribed masses by DBT improves the accuracy of mammography interpretation and may decrease the recall rate in mammography screening.

